# Miscellaneous

**Published:** 1892-11

**Authors:** 


### MISCELLANEOUS.

### SPIRIT HELP.

Speaking of ghosts, said Mr. C. M. Convers, at the Lindell, I must say that they treated me well. I owe all my prosperity to them. In 1866 my wife's father died, leaving, so far as we knew, no property. After his demise my wife declared that he frequently appeared to her and always in the daytime. She was much frightened, and insisted upon moving into another house. We moved, but the apparition was not to be got rid of thus. She declared that it always looked as though it wanted to speak to her, and I advised her to encourage it to unfold the secrets of its prison house.

One day it appeared to her as she was placing dinner on the table, and she mustered up courage to ask it what it wanted. It replied that many years before death my wife's father had acquired several town lots in Philadelphia, which are now quite valuable, and that the deed to the same could be found in an old copy of "Plutarch's Lives," of which he was very fond. We hunted up the book, and sure enough, there was the deed. My wife was the only heir, and the message from the dead was worth a cool $20,000 to us. Now, I did not see the ghost, and do not know whether my wife saw it or simply imagined it. I cannot say whether the message was conveyed by word of mouth, or by some mysterious spirit telegraphy, but I do know that I found the deed as directed and got possession of the property. It was an honest ghost.—*St. Louis Globe-Democrat.*

### CURIOUS TREES.

There are many vegetable wonders in this world of ours. Certain tropical trees furnish clothes as well as food, and the inner bark of others is smooth and flexible enough to serve as writing paper. The bread tree has a solid fruit, a little larger than a cocoa-nut, which, cut in slices and cooked, can scarcely be distinguished from excellent bread. The weeping tree of the Canary Islands is wet, even in a drought constantly distilling water from its leaves ; and the wine trees of Mauritius Island furnishes good wine instead of water. A kind of ash in Sicily has a sap which hardens into crude sugar, and is used as such by the natives, without any refining. The product of the wax tree of the Andes resembles beeswax very closely. Then there is the butter tree of Africa, which produces as much as a hundred pounds at once, only to be renewed in a few months. This secretion, when hardened and salted, is difficult to distinguish from fresh, sweet butter. Closely rivaling this is the milk tree of South America, the sap of which resembles rich cow's milk, and is used as such by the natives. China can boast of a soap tree, the seeds of which when used as soap produce strong suds, and remove dirt and grease readily. In direct opposition to these useful trees is the man-eating plant of the tropics, which resembles Venus's fly-trap in its nature. It has a short, thick trunk armed with narrow flexible barbed spines.

How the Lucifer Match was Invented.—It is not generally known that it is to Isaac Holden, M. P., that we owe the invention of the lucifer match. The discovery was, he has told us himself, the result of a happy thought. In the morning I used to get up at four o'clock in order to pursue my studies, and I used at that time the flint and steel, in the use of which I found very great inconvenience. Of course I knew, as other chemists did, the explosive material that was necessary in order to produce instantaneous light, but it was very difficult to obtain a light on wood by that explosive material, and the idea occurred to me to put sulphur under the explosive mixture. I did that and showed it in my next lecture on chemistry, a course of which I was delivering at a large academy. There was, said Mr. Holden, a young man in the room whose father was a chemist in London, and he immediately wrote to his father about it, and shortly afterward lucifer matches were issued to the world. I believe that was the first occasion that we had the lucifer match. I was urged to go and take out a patent immediately, but I thought it was so small a matter and it cost me so little labor that I did not think it proper to go and get a patent, otherwise I have no doubt it would have been very profitable.—*Pall Mall Gazette.*

Tea Chest Lead.—One of the industries in connection with the tea trade is the collection of the lead with which tea chests are lined. China has been noted for many centuries for the purity of its lead, and this tea-chest lead, as it is called, is regarded as the finest in existence. There are many uses for it; it is found very valuable in making the best kind of solder. No machinery is employed in the production of this sheet lead; every sheet is made by hand in the most primitive fashion. A large brick is provided, the size of the sheet of lead to be made, and is covered with two or three sheets of paper. On these the molten lead is poured, and another brick is placed on the top, which flattens the lead out the required size and thickness. The sheets are then soldered together to the size of the interior of the tea chest; the tea is packed in; and the top sheet is fastened in place. The workmen are very expert, and they turn out an immense number of sheets in the course of a day, and, where labor is so cheap, at a price much less than if the articles were produced by machinery.

Fruit Canning.—Few people are aware that we are indebted to the people of old Pompeii, who were all smothered in the first century of the Christian era, for one of the most important industries of our time—the canning business. Years ago, when the first excavations were made in that buried city, an American came upon several jars of figs. When they were opened the contents were found to be as fresh and palatable as when they were put up eighteen centuries before. Investigations instituted on the spot proved that the fruit had been put into jars in a highly heated state, and that an aperture for the escape of steam had been left in the lid, which, when it had served its purpose, was sealed over with wax. Yankee ingenuity caught the idea at once and the next year canning factories were erected all over the United States.

Storms.—The rate of progression of a storm is often fifty miles an hour, and a series has often been traced in a direct line from north to south a distance of 400 miles. The average altitude of thunderstorms has been found to be not over 5,000 feet above the surface of the earth.

IMPORTANT.

Distinguished contributors to the medical press assert that many cases of skin diseases are originated, and others indefinitely kept up, by the use of soaps made from impure fats. They advise the exclusive use of Vegetable Oil Soaps, and commend particularly Packer's Tar Soap, which is made from vegetable oils, glycerine and pine tar.

